# Application of surface-enhanced Raman spectroscopy in the diagnosis and treatment of breast cancer

**DOI:** 10.3389/fmed.2025.1725221

**Published:** 2026-01-09

**Authors:** Xin-Xin Luo, Meng-Ye He, Meng-Li Luo, Yun-Lu Jia, Peng Shen

**Affiliations:** 1 Department of Internal Medicine, Yuyao Maternity and Child Health Care Hospital, Ningbo, Zhejiang, China; 2 Department of Medical Oncology, The First Affiliated Hospital, Medical College of Zhejiang University, Hangzhou, Zhejiang, China

**Keywords:** breast cancer, early detection, liquid biopsy, surface-enhanced Raman spectroscopy, tumour diagnosis

## Abstract

Current tumour diagnosis relies on patient symptoms, physical signs, and imaging findings supplemented by the gold standard, pathological examination. Posttreatment efficacy evaluation also primarily depends on imaging aided by blood tests, such as tumour marker tests, which often require invasive procedures. In recent years, scientists have explored more sensitive and noninvasive tumour diagnostic technologies. Owing to its single-molecule level sensitivity, fingerprint-like specificity, synchronous multicomponent detection capability, noninvasiveness, and potential for rapid analysis, surface-enhanced Raman scattering (SERS) technology has gradually gained attention in the medical field, particularly for tumour diagnosis and treatment. This article systematically reviews SERS detection for breast cancer diagnosis and therapy.

## 1 Introduction

Breast cancer, one of the most prevalent malignancies worldwide, requires early diagnosis as a critical determinant for improving the five-year survival rate of patients (1). Current clinical diagnostic methods are often limited by their invasive nature, operational complexity, and prolonged processing time (2). Against the backdrop of growing demands for molecular subtype-guided precision therapy, conventional detection techniques face significant challenges in achieving highly sensitive detection of minute biomarkers such as circulating microRNAs (miRNAs). Traditional tumour diagnostic methods, such as imaging examinations and tissue biopsies, are widely used in clinical practice but still exhibit limitations in early tumour detection, primarily due to insufficient sensitivity for identifying early-stage abnormalities. Imaging techniques such as CT involve ionizing radiation, whereas ultrasound (3), although applicable to most patients, suffers from low resolution, making it difficult to detect small lesions. MRI offers high resolution without radiation but requires extremely strong magnetic fields and is contraindicated for patients with pacemakers (4). Imaging examinations may also require contrast agents, posing risks for patients with renal insufficiency or contrast allergies. Tissue biopsies are invasive, risky, expensive, painful, and not always feasible. Furthermore, aggressive subtypes such as triple-negative breast cancer frequently exhibit early metastatic behavior, for which conventional imaging modalities demonstrate significant limitations in detecting micrometastases (5). The precise intraoperative delineation of tumor margins also remains a major challenge, with approximately 30% of patients undergoing breast-conserving surgery experiencing postoperative recurrence due to residual tumor tissue (6). These issues collectively underscore critical bottlenecks in current diagnostic systems regarding sensitivity, specificity, and real-time capability.

SERS technology utilizes plasmonic nanostructures to achieve orders-of-magnitude enhancement of Raman signals from adsorbed molecules, amplifying molecular vibration information through electromagnetic field mechanisms and enabling detection sensitivity at the single-molecule level (7, 8). The technical advantages of SERS are threefold: first, its ultra-high specificity with distinctive “molecular fingerprint” characteristics enables direct identification of tumor-derived exosomes through characteristic spectral profiles (9); second, its non-invasive detection capability permits analysis with minimal volumes of bodily fluids (10); third, its potential for multiparameter synchronous detection allows simultaneous capture of multiple breast cancer-associated miRNAs using substrate materials such as seed-mediated grown silver nanopillars (SMGAPs) (1). Compared with conventional Raman spectroscopy, SERS exhibits superior photobleaching resistance (7), and when combined with gap-enhanced Raman nanotags, enables simultaneous discrimination of at least three breast cancer surface protein biomarkers (11). These unique characteristics make SERS particularly valuable in circulating tumor cell detection (1) and intraoperative navigation (12).

The translation of SERS technology from basic research to clinical application represents a paradigm shift in cancer diagnostics and treatment (13). In diagnostic applications, its integration with microfluidic chips enables label-free detection of plasma exosomes (14), while artificial intelligence-assisted spectral analysis has achieved 100% accuracy in breast cancer diagnosis (15). In the therapeutic domain, SERS-guided surgical systems allow precise tumor margin delineation (6), and “sandwich” structured sensors based on gold nanorod arrays offer novel approaches for detecting metabolic biomarkers such as serotonin (16). The clinical implementation of SERS technology will propel breast cancer management into the era of “real-time molecular subtyping” (5), ultimately establishing an integrated diagnostic-therapeutic-prognostic solution through the combination of exosome analysis (9) and drug delivery monitoring (17). This multimodal theranostic approach (14) holds significant promise for addressing critical clinical challenges, including narrow early detection windows and difficult postoperative recurrence monitoring in breast cancer.

## 2 Research advances in SERS technology for breast cancer diagnosis

**Figure 1:**
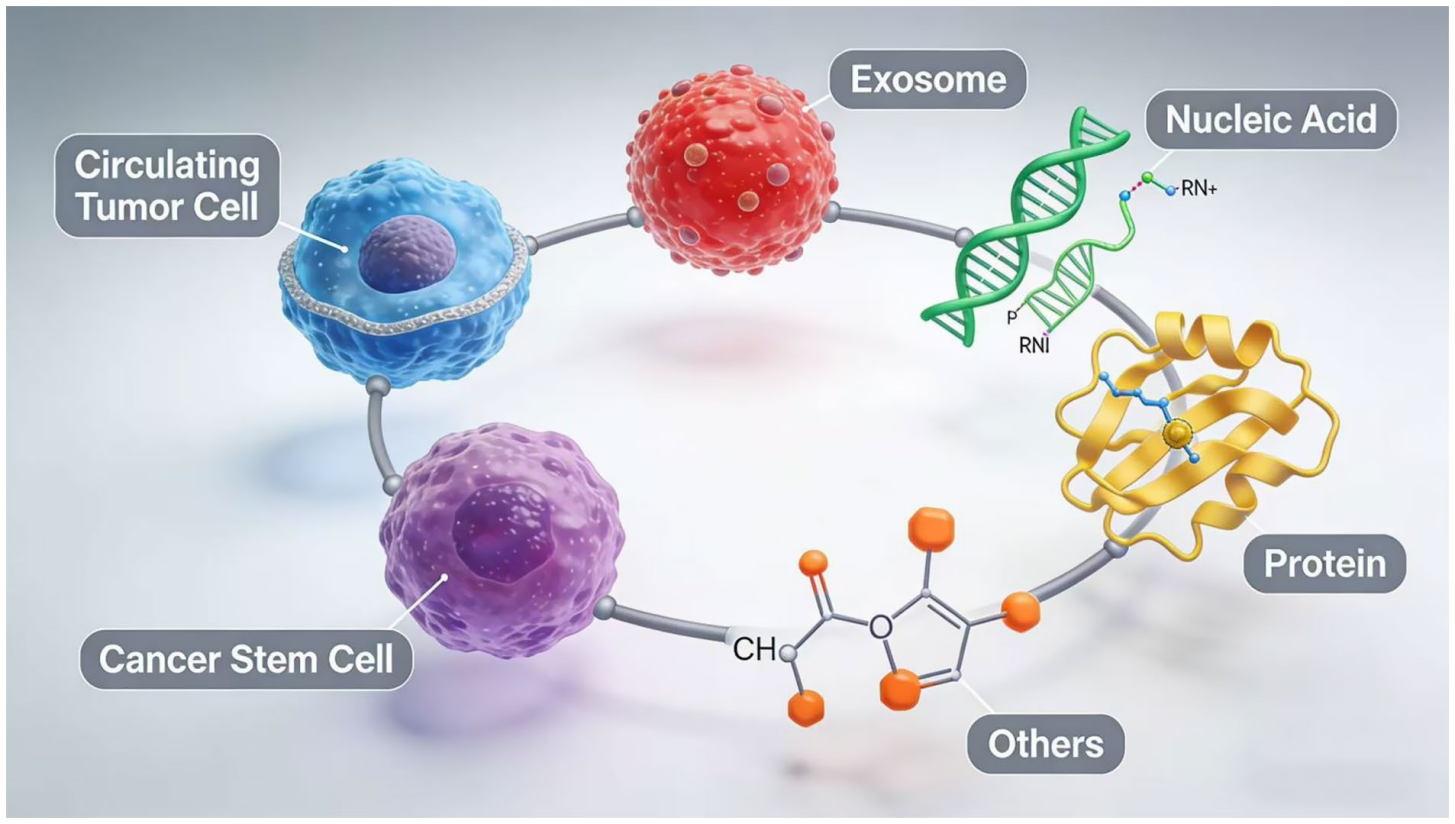
Common directions of SERS in diagnosis and treatment of breast cancer.

SERS has demonstrated significant advantages in breast cancer biomarker detection owing to its exceptional sensitivity and unique molecular fingerprinting capabilities. The conventional single-marker detection paradigm is progressively evolving into integrated multi-omics strategies, which substantially enhance diagnostic accuracy by simultaneously capturing information from diverse biomolecules including proteins, nucleic acids, and metabolites. Particularly when combined with deep learning algorithms, SERS enables precise molecular subtyping of breast cancer through extraction of multi-omics features from complex spectral data. This multi-analyte detection approach effectively overcomes the limited specificity inherent in single-biomarker assays, providing novel pathways for developing more reliable diagnostic models for breast cancer. At present, the common direction of SERS technology in the diagnosis and treatment of breast cancer is shown in Figure 1.

### 2.1 Liquid biopsy applications: circulating tumor cell and exosome analysis

As the fundamental structural and functional units of living organisms, cells communicate through signaling mechanisms that regulate critical biological processes including proliferation, differentiation, and apoptosis. These cellular interactions are intimately associated with disease pathogenesis and progression (18). This fundamental characteristic establishes cells as valuable biomarkers for disease diagnosis (19). Among various cellular diagnostic technologies, the detection of circulating tumor cells (CTCs) has gained particular prominence as a representative indicator in the liquid biopsy field.

CTCs originate from primary tumor tissues, enter circulation through vascular or lymphatic systems, and ultimately establish metastatic foci in distant organs (20). Analysis of their numerical and phenotypic characteristics holds significant importance for the diagnosis and treatment of malignant tumors. Current CTC-based detection systems have received FDA approval for monitoring disease progression and therapeutic efficacy in metastatic breast cancer (21–24). Notably, CTCs exist in extremely low concentrations (1–10 cells per milliliter of blood) amidst overwhelming numbers of blood cells (millions of leukocytes and billions of erythrocytes) (25). This substantial quantitative disparity presents a fundamental challenge for current cellular diagnostic technologies: the efficient capture and precise analysis of rare cells from complex blood samples.

SERS technology demonstrates unique value in liquid biopsy by enabling detection of various circulating biomarkers, including CTCs and exosomes (26). Zhang et al. (27) developed a magnetic Fe_3_O_4_@Au composite SERS bioprobe capable of effectively capturing triple-negative breast cancer (TNBC) cells while endowing them with distinctive SERS signatures. By comparing SERS signal intensities between TNBC cells and white blood cells (WBCs), they established a tumor cell identification model with the unique advantage of intelligent switching between detection sensitivity and specificity for different identification purposes (such as precise diagnosis versus preliminary screening). Finally, through spiking TNBC cells into healthy human blood and testing genuine patient-derived blood samples, they validated the model’s dual-function capability for both precise diagnosis and initial screening. This innovative strategy provides fresh perspectives for CTC identification via SERS technology.

For exosome detection, SERS enables label-free acquisition of their unique molecular fingerprints, which reflect the pathological status of parent cells (28). Research demonstrates that using 3D plasmonic gold nano-film substrates combined with machine learning algorithms, SERS achieves 93.3–100% accuracy in exosome detection (4, 29). Lei et al. (30) successfully constructed a DNA aptamer-based SERS probe with silver-coated gold nanorods (Au@Ag@IDA-B/4MSTP) for detecting integrin α6β4 in breast cancer-derived extracellular vesicles (EVs)—a biomarker demonstrated to directly correlate with breast cancer progression. This probe achieved a detection limit of 23 particles/μL for EVs derived from cell cultures, with its reliability validated in mouse models of subcutaneous tumors and lung metastasis. Using only 10 μL of plasma samples, the method attained 85.7% sensitivity and 83.3% specificity, while significantly streamlining the detection process. This non-invasive methodology offers novel approaches for early breast cancer diagnosis and postoperative monitoring (31).

### 2.2 Genomic applications: nucleic acid, protein and cancer stem cell detection

In genomic testing, SERS technology enables ultrasensitive detection of nucleic acid biomarkers such as breast cancer-associated microRNAs. By employing specifically designed nanoprobes, SERS can identify nucleic acid sequences with single-base discrimination, achieving the sensitive detection of miRNA-21 down to the 0.1 fM level in a linear range of 10 fM to 1 nM (32). Xu et al. (33) developed an Au@Ag core-shell nanorod-based SERS sensor incorporating dual-specific nuclease-mediated signal amplification. This system simultaneously quantifies three breast cancer-related miRNAs (miR-21, miR-155, and let-7b) by monitoring Raman signal attenuation at hot spots in the presence of target miRNAs, with remarkably low detection limits (LOD) of 0.05 fM, 0.063 fM, and 0.037 fM, respectively, thereby contributing to early breast cancer diagnosis.

Furthermore, SERS demonstrates significant utility in detecting key protein biomarkers including HER2, ER, and progesterone receptor(PR). Murali et al. (34) developed a Raman-label-based surface-enhanced Raman scattering (RL-SERS) nanoprobe technology that enables simultaneous detection of multiple biomarkers in heterogeneous breast cancer tissues. This technology constructs RL-SERS nanotags capable of concurrently identifying ER, PR, and HER2 by sequentially modifying gold nanoparticles with characteristic Raman labels and specific antibodies. Utilizing a ratiometric characteristic Raman signal analysis method, the study successfully achieved synchronous detection of single, dual, and triple biomarkers in clinically confirmed formalin-fixed paraffin-embedded (FFPE) breast tissue samples, effectively reducing false negatives and false positives. Utilizing gap-enhanced Raman nanotags combined with machine learning algorithms, Rodriguez-Nieves et al. (11) effectively detected and classified surface proteins associated with three distinct molecular subtypes of breast cancer, enabling precise molecular subtyping Khosroshahi et al. (35) developed a label-free SERS immunosensor based on plasmon-activated nanostructured films capable of detecting HER-2 overexpression (2+, 3+, and positive) in patient serum. This dual capability for concurrent nucleic acid and protein detection establishes SERS as a powerful tool for investigating the relationship between genetic variations and protein expression in breast cancer.

The application of SERS technology in cancer stem cell research shows equally promising prospects. Haldavnekar et al. (36) developed a nickel-based functionalized nanoprobe SERS technique for monitoring cancer stem cells (CSCs) and prognostic prediction. This technology successfully distinguishes molecular characteristics between CSCs and ordinary cancer cells across various cancer cell lines (including breast, cervical, and lung cancers) through single-cell sensitivity SERS analysis, while precisely monitoring the intermediate states of mutual transformation between CSCs and cancer cells. The key breakthrough lies in the discovery that quasi-intermediate states in different transformation directions possess unique molecular signatures, which enables approximately 99% accurate prediction of cancer dissemination and provides a novel tool for ultrasensitive prognosis of malignant tumors.

### 2.3 Nanoprobe development: targeted recognition and molecular subtyping

The development of targeted nanoprobes represents a critical breakthrough in applying SERS technology to breast cancer diagnosis. By engineering multicolor nanoprobes incorporating distinct Raman reporter molecules, simultaneous identification of specific protein markers on breast cancer cell surfaces can be achieved. These probes typically employ core-shell architectures, such as magnetic core-gold shell nanoparticles, which not only enhance Raman signals but also facilitate target molecule enrichment through magnetic field manipulation (11). Recent advancements in gap-enhanced nanostructures have further elevated detection sensitivity to the single-molecule level (37), establishing a foundation for precise molecular subtyping of breast cancer.

### 2.4 Microfluidic chip integration: automation and high-throughput detection

The integration of microfluidic technology with SERS has significantly enhanced both automation and detection throughput in breast cancer diagnostics. These integrated systems enable complete automation of the entire workflow, including sample pretreatment, target molecule capture, and SERS detection, substantially streamlining operational procedures (13). Furthermore, microfluidic chip designs allow precise control over interaction times between nanoprobes and target molecules, thereby improving detection reproducibility (38). Current systems can achieve high-throughput analysis of dozens of samples per hour, providing crucial engineering solutions for the clinical translation of SERS technology (39).

## 3 SERS guided treatment strategy for breast cancer

### 3.1 Intraoperative tumor margin delineation

Breast-conserving surgery has become the preferred therapeutic option for breast cancer patients owing to its minimal invasiveness and superior cosmetic outcomes. However, incomplete resection of microscopic lesions during surgery often leads to postoperative local recurrence. To address this challenge, Wen et al. (40) developed an intraoperative navigation strategy based on SERS. This approach utilizes a multifunctional SERS probe targeting HER2, which integrates ultra-sensitive detection, HER2 expression suppression, cell proliferation inhibition, and efficient photothermal therapy. This system enables precise delineation of tumor margins while providing real-time ablation of residual microscopic foci, thereby ensuring complete tumor resection and significantly improving surgical outcomes. In a HER2-positive breast cancer mouse model, the SERS-navigated surgical system successfully achieved complete tumor resection combined with intraoperative real-time photothermal ablation. Experimental results demonstrated complete tumor eradication without local recurrence in all treated animals, yielding a 100% tumor-free survival rate. Wen et al. (41) developed a multifunctional theranostic nanoprobe based on gold nanostars (starPART), achieving intraoperative image-guided tumor resection and SERS-navigated photothermal eradication of residual microtumors. This probe seamlessly integrates three key functionalities: photoacoustic imaging, SERS detection, and photothermal therapy. Preoperatively, it enables tumor margin delineation through photoacoustic imaging to guide surgical resection; intraoperatively, it utilizes SERS technology for real-time identification and precise localization of residual lesions while simultaneously performing photothermal ablation. Animal studies have confirmed that this strategy achieves complete eradication of microtumors, resulting in 100% tumor-free survival without local recurrence. This SERS-guided surgical strategy holds considerable promise as a novel clinical approach for HER2-positive breast cancer, with the potential to substantially improve patient survival outcomes.

### 3.2 Molecular subtyping of tumors *in vivo* and *in vitro*

**Figure 2:**
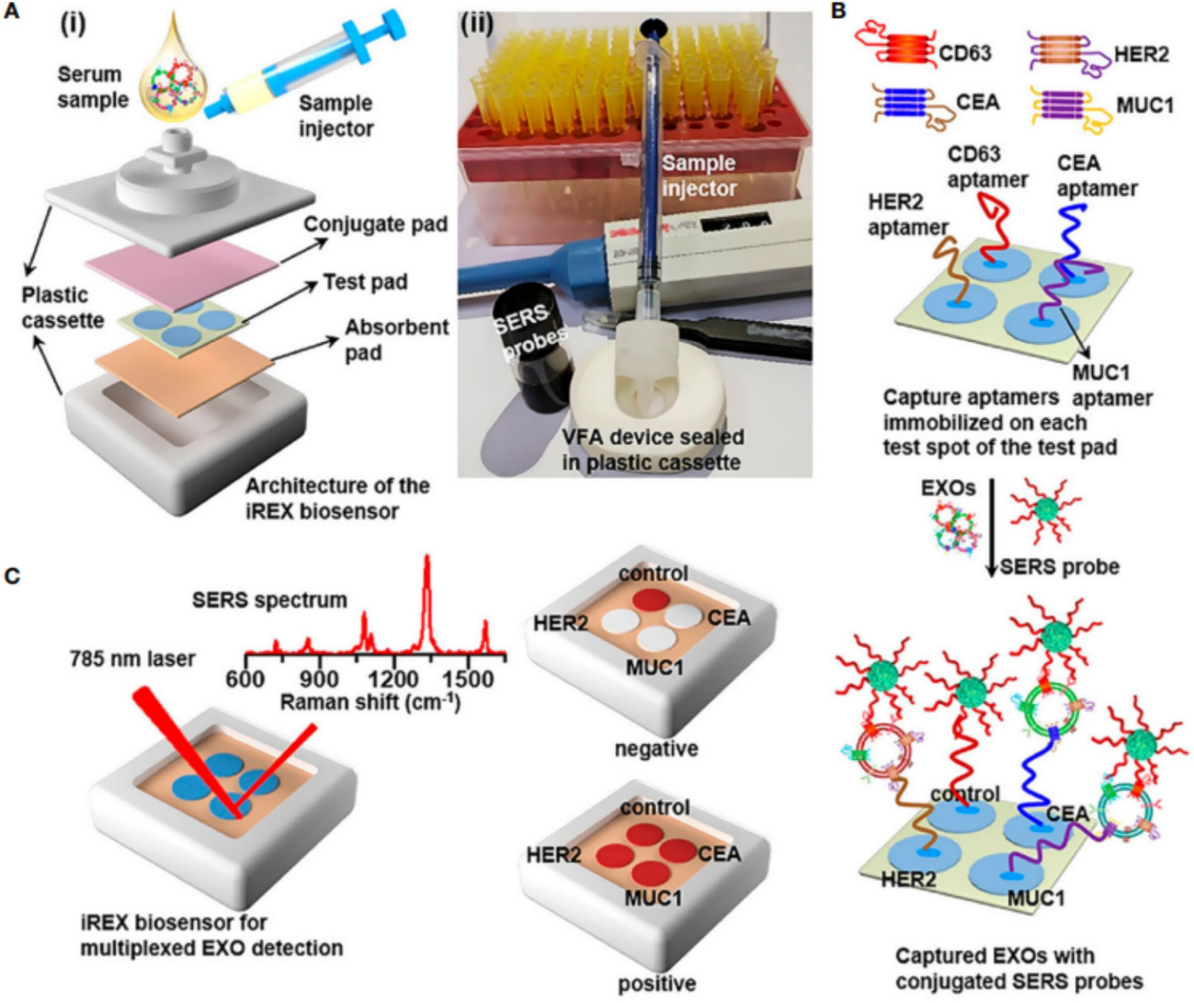
**(A)** (i) Overview of the architecture (i) the real image of the iREX biosensor (ii). **(B)** Illustration of multiplexed detection of exosomal proteins in serum samples. **(C)** Schematic of the iREX biosensor based multiplexed detection of exosomal proteins in the presence of no EXOs (negative test) and multiple EXOs (positive test).

SERS technology, when integrated with multicolor Raman tags and machine learning algorithms, enables high-throughput identification of molecular subtypes in breast cancer. By employing core-shell nanostructured tags incorporating distinct Raman reporter molecules, this approach allows simultaneous detection of three breast cancer-specific surface protein biomarkers (11). This molecular subtyping method not only achieves single-cell level detection sensitivity but also rapidly acquires molecular characteristics during surgical procedures, thereby providing critical information for immediate treatment decisions. Compared with conventional immunohistochemistry, SERS-based subtyping significantly reduces processing time (from several days to hours) while avoiding epitope damage during tissue processing (42). Xie et al. (15) reported an artificial intelligence assisted SERS strategy for unlabeled spectral analysis (Figure 2). By using the deep learning algorithm of SERS spectral training of cancer cell derived exosomes, the SERS spectrum of serum exosomes is used to achieve 100% prediction accuracy in patients with different breast cancer subtypes. Zhang et al. (2) developed a novel optical detection method integrating SERS with LightGBM-deep neural network algorithms for non-invasive screening and molecular subtyping of breast cancer through serum component analysis. The study collected serum SERS spectra from breast cancer patients, patients with benign breast diseases, and healthy controls. After employing the LightGBM algorithm to identify key spectral features, a diagnostic model was constructed using deep neural networks. This approach achieved 91.38% accuracy in distinguishing breast cancer from benign lesions, and reached 96.40% accuracy in the three-class classification task (breast cancer/benign lesions/healthy controls). For molecular subtyping, the method attained 90.11% accuracy in differentiating hormone receptor status and 88.89% accuracy in distinguishing HER2 status. Su et al. (43) developed an iREX biosensor based on paper-based SERS-vertical flow detection technology, achieving multiplexed quantitative profiling of breast cancer exosomal proteins. By simultaneously detecting the expression profiles of three proteins (MUC1, HER2, and CEA) in exosomes, this technology successfully distinguishes different molecular subtypes of breast cancer. The study validated the clinical utility of this approach using clinical serum samples, demonstrating its high sensitivity, molecular specificity, and multiplex detection capability, thereby providing a novel clinical tool for non-invasive subtyping and treatment monitoring of breast cancer.

### 3.3 Real-time monitoring of drug delivery systems

SERS technology offers unique capabilities for monitoring *in vivo* delivery of antitumor drugs. By labeling drug carriers with Raman-active molecules, real-time tracking of nanodrug accumulation at tumor sites becomes feasible. The electrically enhanced adsorption combined with SERS technique (EE-SPME-SERS) enables monitoring of drug release kinetics with second-level temporal resolution, a feature of significant importance for optimizing dosing regimens (17). Furthermore, modular SERS labeling strategies that precisely control the number of Raman reporter molecules significantly enhance detection sensitivity (limit of detection: 10^−18^ M), creating opportunities for precise monitoring of low-dose drug delivery.

### 3.4 Combined photothermal-photodynamic therapy

SERS-active nanomaterials (such as gold nanoshells) can simultaneously function as photothermal conversion agents and Raman enhancement substrates, achieving integrated diagnosis and therapy. Under near-infrared laser irradiation, the localized surface plasmon resonance effects generated by these nanomaterials not only enhance Raman signals for tumor imaging but also induce photothermal effects for tumor ablation (44). Dual-modal SERS imaging strategies (e.g., combined with photoacoustic imaging) further improve tumor localization accuracy during treatment, particularly for targeted therapy of refractory subtypes like triple-negative breast cancer (7). This combined therapeutic approach demonstrates significant tumor suppression in animal models while minimizing damage to surrounding normal tissues (7, 44).

### 3.5 Multimodal theranostic integration: combining with other diagnostic and therapeutic approaches

The integration of SERS technology with multiple imaging modalities has established a new paradigm for multimodal theranostics in breast cancer. Surface-enhanced spatially offset Raman spectroscopy (SESORS) overcomes the tissue penetration limitations of conventional Raman techniques, enabling non-invasive localization of deep-seated sentinel lymph nodes (45). Transmission Raman spectroscopy (TRS) systems combined with ultra-bright SERS nanoparticles provide real-time intraoperative navigation for deep tumor resection, achieving picomolar-level detection sensitivity (46). Additionally, diagnostic strategies integrating SERS with machine learning algorithms significantly enhance diagnostic accuracy (achieving 100% prediction accuracy) through simultaneous analysis of multiple tumor biomarker fingerprint spectra (47, 48). Hou et al. (49) established a multimodal predictive model integrating serum SERS spectroscopy with clinicopathological features, achieving 90% accuracy (AUC = 0.93) in independent validation, which effectively distinguishes major responders from non-major responders to neoadjuvant therapy. By identifying serum biomarkers including uric acid and tryptophan, the model provides molecular evidence for predicting treatment response. The clinical utility of this technology is demonstrated through: early efficacy prediction during treatment to avoid ineffective therapies; simple operation requiring only serum samples; and superior performance compared to single-modality detection methods, providing a reliable tool for developing individualized treatment plans. These multimodal systems provide comprehensive technical support for early diagnosis, precision treatment, and efficacy evaluation in breast cancer management.

## 4 Discussion

Although SERS technology has been applied in the whole process of diagnosis and treatment of breast cancer, there is still much room for development. Based on its development limitations and potential innovative directions, we will make the following discussion.

### 4.1 Four major challenges in clinical translation

#### 4.1.1 Need for device miniaturization and operational simplification

The primary challenge in the clinical translation of SERS technology lies in the bulky equipment and complex operational procedures. Conventional SERS systems require precise optical components and stable experimental environments, which contrast sharply with the clinical demand for portable and user-friendly devices. Particularly in intraoperative settings for breast cancer, existing equipment struggles to meet spatial constraints and real-time detection requirements in operating rooms (40). Although integration of microfluidic chips with SERS has been attempted to achieve automated detection (1), the overall system integration and operational convenience still fall short of clinical practicality.

#### 4.1.2 Lack of standardized detection and quality control systems

The absence of unified detection standards and quality control systems represents another critical bottleneck hindering the clinical application of SERS technology. Since enhancement effects vary with nanostructure morphology, laser power, and other factors, significant discrepancies often exist between results obtained from different laboratories. In breast cancer biomarker detection, such variability directly impacts the reliability of key clinical decisions such as HER2 status assessment. Although artificial intelligence algorithms have been introduced to improve spectral analysis consistency (50), internationally accepted protocols for key steps including sample preprocessing and signal acquisition conditions remain underdeveloped.

#### 4.1.3 Bottleneck in multicenter reproducibility

Insufficient reproducibility across multiple centers severely restricts the clinical validation process of SERS technology. Reported diagnostic accuracy for breast cancer varies, primarily due to heterogeneity in substrate materials, detection protocols, and data analysis methods across institutions. Particularly in SERS-based exosome detection, variations in sample preprocessing methods can alter surface protein conformations, consequently affecting spectral characteristics. The lack of standardized sample preparation protocols prevents direct comparison of data from different research centers.

#### 4.1.4 Insufficient large-scale clinical validation data

The most prominent obstacle to clinical translation is the scarcity of large-scale population validation data. Although studies have demonstrated the potential of SERS for early diagnosis of certain cancers (51), dedicated validation studies for breast cancer generally involve limited sample sizes (typically <200 cases) and lack long-term follow-up data. While SERS nanoprobes have enabled intraoperative tumor margin delineation, their value in predicting recurrence after breast-conserving surgery remains unvalidated by multicenter cohort studies. Furthermore, existing clinical research predominantly focuses on diagnostic performance evaluation, with systematic investigation of key translational medicine parameters such as cost-effectiveness and health economic indicators remaining largely unexplored.

### 4.2 Technological optimization and innovation directions

#### 4.2.1 Development of novel substrate materials

In surface-enhanced Raman spectroscopy, the performance optimization of substrate materials is crucial for improving detection sensitivity and specificity. Recent advances in gap-enhanced nanostructures, such as magnetic core-gold shell-based gap-enhanced Raman nanotags, have demonstrated significant advantages. These materials maximize electromagnetic field enhancement by incorporating precisely controlled nanogaps (typically <10 nm), achieving enhancement factors ranging from 10^8^ to 10^10^. Studies have shown that anisotropic magnetic core-gold shell gap-enhanced nanotags, when combined with multiple Raman reporter molecules, enable multicolor discrimination of specific protein biomarkers on breast cancer cell surfaces (11). Nevertheless, further exploration of novel substrate materials remains essential.

#### 4.2.2 Artificial intelligence-assisted spectral analysis

Artificial intelligence provides breakthrough solutions for deciphering complex SERS spectral data. Deep learning algorithms trained on SERS spectral databases of cancer cell-derived exosomes facilitate label-free spectral analysis for breast cancer diagnosis. In molecular subtyping, SERS optical detection methods integrating feature selection algorithms and deep learning can extract key biomarker information from complex spectra, significantly enhancing the efficiency and accuracy of breast cancer subtype classification (52). Machine learning-driven SERS platforms also enable dynamic monitoring of HER2 expression levels, demonstrating high linear correlation (R^2^ = 0.9816) with conventional immunohistochemical analysis (2). To address challenges posed by limited sample sizes, novel computational frameworks such as Contextual Explainable AI Recursion (CEAIR) integrate computer vision and cooperative game theory to extract domain-relevant digital biomarkers from high-dimensional SERS data of limited serum samples (53).

#### 4.2.3 Construction of multimodal detection systems

Multimodal integration represents a crucial direction for enhancing the clinical applicability of SERS technology. The combination of SERS with microfluidic technology enables automated, high-throughput detection of circulating tumor cells and exosomes. For *in vivo* imaging, PEG-modified gold nanoparticles serve dual functions as CT contrast agents for precise three-dimensional reconstruction of liver architecture and as SERS-active substrates for simultaneous detection of hepatic injury (54). Advanced SERS-photoacoustic-fluorescence trimodal probes leverage synergistic enhancement effects to concurrently provide molecular fingerprint information, deep-tissue imaging, and real-time dynamic monitoring capabilities (55). Furthermore, the introduction of SESORS enables penetration depths exceeding 50 mm, creating new possibilities for *in situ* detection in deep tissues such as the breast (2). These multimodal systems, through their complementary advantages, are advancing the transition of SERS from a standalone detection technique to an integrated theranostic platform.

### 4.3 Recommendations for clinical translation pathways

#### 4.3.1 Tiered validation system: from laboratory to phase III clinical trials

The clinical translation of SERS technology necessitates the establishment of a systematic tiered validation framework. At the laboratory stage, technical principle verification and *in vitro* model testing must be completed, including validation of SERS spectral data trained with artificial intelligence algorithms achieving 100% predictive accuracy. Subsequently, the preclinical research phase should focus on verifying the biosafety of nanoprobes and their efficacy in animal models, particularly regarding detection performance for circulating tumor cells and exosomes. Phase I clinical trials should evaluate the safety and fundamental performance parameters of SERS detection systems in human subjects. Phase II trials require establishing multicenter cohorts to validate the diagnostic efficacy across diverse populations. Ultimately, Phase III trials should conduct large-scale randomized controlled studies comparing SERS technology with existing standard diagnostic methods.

#### 4.3.2 Establishment of regulatory standards and industry consensus

The clinical application of SERS technology urgently requires unified regulatory standards and industry consensus. Primary needs include developing safety assessment specifications for nanomaterials, particularly biocompatibility standards for metallic substrate materials. Secondly, standardized SERS detection protocols should be established, encompassing unified procedures for sample processing, spectral acquisition, and data analysis. Concurrently, interdisciplinary expert consensus panels should be formed to develop technical guidelines and quality control systems for SERS-based breast cancer diagnosis. Furthermore, regulatory agencies need to establish performance evaluation frameworks for SERS devices, including verification requirements for core metrics such as sensitivity, specificity, and reproducibility.

#### 4.3.3 Cost-effectiveness analysis and medical insurance payment strategies

Promoting the clinical translation of SERS technology requires comprehensive health economic evaluations. Cost analyses should encompass multiple factors including equipment investment, consumable expenses, and personnel training, with particular consideration given to scaled production costs of novel substrate materials such as gap-enhanced structures. Benefit assessments should quantify treatment cost savings and survival benefits resulting from early diagnosis. Medical insurance payment strategies could be implemented in phases: initially as supplemental diagnostic technologies reimbursed per procedure, and subsequently incorporated into diagnosis-related group payment systems as the technology matures. Additionally, innovative payment models should be explored, such as performance-based reimbursement tied to diagnostic accuracy, acknowledging the efficiency improvements enabled by AI-assisted SERS spectral analysis. Multicenter health technology assessments are also necessary to provide evidence-based support for insurance decision-making.

## 5 Conclusion

SERS technology is poised to reshape breast cancer management across three dimensions: diagnostically, its ultra-high sensitivity and molecular specificity will drive the transition from histomorphological criteria to molecular characteristic-based standards; therapeutically, precise intraoperative tumor margin delineation combined with photothermal-photodynamic therapy can significantly reduce recurrence risks in breast-conserving surgery; and in monitoring, exosome-based liquid biopsy enables early recurrence warning. The core value of this transformation lies in overcoming the limitations of conventional approaches in temporal resolution (real-time monitoring) and spatial precision (single-cell level). With the maturation of multimodal detection systems, SERS technology is expected to become a cornerstone methodology throughout the entire continuum of breast cancer care, ultimately realizing the paradigm shift from “population-based therapy” to “personalized intervention.”
